# Boosted-SpringDTW for Comprehensive Feature Extraction of PPG Signals

**DOI:** 10.1109/OJEMB.2022.3174806

**Published:** 2022-05-12

**Authors:** Jonathan Martinez, Kaan Sel, Bobak J. Mortazavi, Roozbeh Jafari

**Affiliations:** Department of Computer Science and EngineeringTexas A&M University 14736 College Station TX 77840 USA; Department of Electrical and Computer EngineeringTexas A&M University 14736 College Station TX 77840 USA; Department of Biomedical Engineering, Electrical and Computer Engineering, Computer Science and EngineeringTexas A&M University 14736 College Station TX 77840 USA

**Keywords:** Dynamic time warping, fiducial point, photoplethysmography, interbeat intervals, wearable sensors

## Abstract

*Goal*: To achieve high-quality comprehensive feature extraction from physiological signals that enables precise physiological parameter estimation despite evolving waveform morphologies. *Methods*: We propose Boosted-SpringDTW, a probabilistic framework that leverages dynamic time warping (DTW) and minimal domain-specific heuristics to simultaneously segment physiological signals and identify fiducial points that represent cardiac events. An automated dynamic template adapts to evolving waveform morphologies. We validate Boosted-SpringDTW performance with a benchmark PPG dataset whose morphologies include subject- and respiratory-induced variation. *Results*: Boosted-SpringDTW achieves precision, recall, and F1-scores over 0.96 for identifying fiducial points and mean absolute error values less than 11.41 milliseconds when estimating IBI. *Conclusion*: Boosted-SpringDTW improves F1-Scores compared to two baseline feature extraction algorithms by 35% on average for fiducial point identification and mean percent difference by 16% on average for IBI estimation. *Significance*: Precise hemodynamic parameter estimation with wearable devices enables continuous health monitoring throughout a patients’ daily life.

## I. Introduction

Hemodynamic parameter estimation with wearable devices enables continuous health monitoring in outpatient settings, thus granting diagnostic insight throughout varying contexts of a patient's daily activities. Not commonly captured in clinical settings, this information contributes to the early detection of life-threatening illnesses, advanced fitness tracking, and emotional regulation [1]. Precise parameter estimation depends on comprehensive feature extraction from a stream of physiological signals continuously captured by conveniently worn wearable devices. Furthermore, features are extracted from distinguishable peaks, valleys, and slopes within the waveform that depict key physiological events. For example, photoplethysmography (PPG) waveform morphology captures blood flow, which may be mapped to heart rate (HR), interbeat interval (IBI), blood pressure (BP), and respiration rate (RR) [2]. However, physiological waveform morphology is highly sensitive to inter-subject and contextual variations that present challenges to the heuristics pre-defined by previously developed feature extraction frameworks [3]. Despite that such variations may still be considered valid representations of cardiac cycles, this variability may lead to inaccurate parameter estimation, ultimately leading to unreliable health diagnoses. In this study, we introduce a comprehensive feature extraction framework that adapts to such morphological variations, ensuring high-quality hemodynamic parameter estimations.

All physiological waveforms that capture blood flow or represent heart beats – including photoplethysmography (PPG) [4], electrocardiography (ECG) [5], and bio-impedance (Bio-Z) [6] – possess a quasi-periodic property corresponding to the contraction and relaxation of the heart. Typical approaches for parameter estimation and analysis, beginning with segmentation, often revolve around hand-crafted fiducial point detection algorithms that are constrained by domain-specific heuristics such as average cycle length or plausible amplitude values [7]–[9]. Unless adaptive thresholding is carefully implemented to consider all possible sources for morphological variation, over time as patients age, vascular health evolves, or as the algorithms are applied to new patients, such methods will fail when faced with a variation that is not considered in the general case. Filtering and transformation of the signals to the frequency domain, such as with Hilbert or wavelet transforms [10]–[12], are more robust, however, they still depend on effective adaptive thresholding for task-specific identification algorithms. Alternatively, machine learning and deep learning [13]–[15] models do not require adaptive thresholding and are robust to waveform variations, but require an abundant labelled training dataset with high waveform variance that is not practical to obtain for patients whose data was not seen in training.

Dynamic time warping (DTW) shows potential for simultaneously identifying all target fiducial points. This established technique compares the likeness between two signals to produce sample-to-sample mappings between them without any prior knowledge on the their underlying physics [16]. Thus, it has been used for subsequence matching, feature extraction, clustering, optimization, and signal quality index (SQI) tasks [17]–[19]. Although it is a good candidate for the objective of this work, in its original form DTW faces two critical challenges when segmenting a stream of data. First, quasi-periodic physiological waveforms contain multiple meta-subsequences that resemble the morphology of a complete cardiac cycle [20], which is detrimental to segmentation tasks since incomplete cycles might be detected. Second, when DTW depends solely on a single template, waveform comparison quality will decline when encountering extreme morphological variations. Although additional innovations have been proposed to encode signal characteristics to a feature vector that may overcome morphological variations [21], these approaches become impractical to determine the optimal feature sets to be used for DTW comparisons.

In this work, we propose Boosted-SpringDTW as an automated approach towards comprehensive feature extraction of physiological waveforms. Particularly, our proposed framework overcomes the aforementioned challenges associated with the two common sub-tasks required for analyzing a stream of quasi-periodic physiological signals: 1) segmentation into cardiac cycles and 2) identification of all fiducial points. Given a single template of a typical cardiac cycle, our framework conducts a probabilistic decision-making process to precisely identify the true start and endpoints within a waveform stream by leveraging both minimum domain-specific characteristics of the physiological signal and the generalizable intuitions of DTW. We also propose a dynamic template that will automatically adapt to new variations without domain expert intervention. Such automated comprehensive feature extraction can contribute to the waveform preprocessing steps required for prediction problems related to remote health monitoring by extracting actionable information from physiological signals collected by wearables.

Our contributions in this paper are summarized as follows:

• We introduce Boosted-SpringDTW which simultaneously identifies all fiducial points of a given waveform stream.
• We propose a probabilistic decision-making process that enhances DTW with minimal domain-specific heuristics, thus enabling the analysis of quasi-periodic signals.
• We incorporate an automated dynamic template to adapt to evolving morphologies.

## II. Materials and Methods

**Fig. 1.**
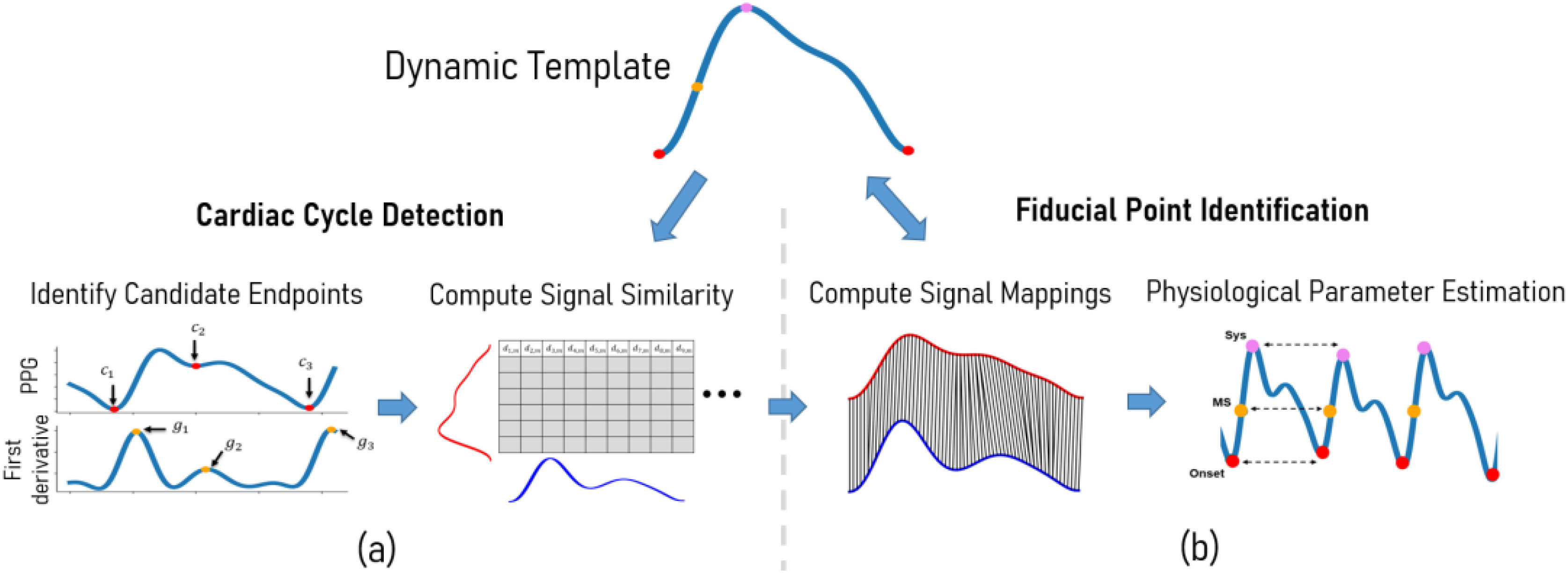
Overview of the proposed Boosted-SpringDTW framework that achieves comprehensive feature extraction through cardiac cycle segmentation and fiducial point identification, a) first we combine domain-specific heuristics with morphology-based comparisons from DTW to achieve cardiac cycle segmentation where ${c_t}$ are candidate endpoints, ${g_t}$ are the max slope amplitudes used to rank candidate endpoints, and ${d_{t,m}}$ is the DTW distance between the stream and template waveforms, and b) we use the DTW sample-to-sample mappings to identify fiducial points – Onset is the cardiac cycle endpoint, MS is the max slope point, and Sys is the systolic peak. We also introduce of a dynamic template that may adapt to evolving morphologies.

Boosted-SpringDTW – shown in Fig. 1 – achieves comprehensive feature extraction through two sub-tasks solved simultaneously: 1) cardiac cycle detection (segmentation) and 2) fiducial point identification. The segmentation task leverages minimal prior domain-knowledge of the target signal to first identify all realistic candidate endpoints for each cardiac cycle. Then, the true endpoints are distinguished by tracking DTW distance scores within a reasonable search space constrained by HR. Fiducial points are simultaneously identified based on the sample-to-sample mappings. New templates are automatically generated over time as the waveform morphology evolves. In this study, we apply Boosted-SpringDTW to streams of PPG waveforms, however, this approach is applicable to all types of physiological waveforms. We validate the effectiveness of our proposed framework when identifying fiducial points for a physiological parameter estimation task with a benchmark PPG dataset whose waveforms are impacted by subject- and respiration-induced variation. The scope of this work includes analysis of valid cardiac cycles’ waveform morphologies; therefore, we exclude those severely corrupted by artifact noise such as those caused by motion.

### A. Cardiac Cycle Detection & Fiducial Point Identification

Segmenting physiological signals into cardiac cycles involves assigning a probability, $P({{e_t}})$ to each sample, ${x_t}$, in the given stream of PPG waveform, $X$. This represents the likelihood in which each sample is either a start or endpoint, ${e_t}$, of a cardiac cycle (segment) computed as

\begin{equation*}
P\left({{e_t}} \right) = P\left({{c_t}|{x_t}} \right)*P\left({d\left({{x_t},{y_m}} \right)} \right) \tag{1}
\end{equation*}

where $P({{c_t}})$ is the likelihood based on the minimal domain-specific heuristics which consider the underlying physics of the waveform where ${c_t}$ represents a candidate endpoint in $X$, and $P({d({{x_t},{y_m}})})$ is the likelihood based on morphology similarity evaluated by DTW where $d({{x_t},{y_m}})$ is the DTW distance between a given sample of X, ${x_t}$, and a given sample of the template waveform $Y$, ${y_m}$. Furthermore, the DTW distances that are typically monitored for subsequence matching in the standard SpringDTW approach are “boosted” with the domain-specific heuristic likelihood score to bring forward a probabilistic decision-making process. We describe the computation of each in the following paragraphs.

We detect the local maxima of a series of likelihood scores to identify the time steps that are the true cardiac cycle endpoints. Local maxima are defined by the dominant frequency, ${f^*}$, of the pulsatile signals, which corresponds to HR. Using a standard Fast Fourier Transform (FFT) [22] to obtain, ${f^*}$, we estimate the average cardiac cycle length, ${l_X}$, for windows of the input batch with:

\begin{equation*}
{l_X} = \ \frac{{{f_s}}}{{{f^*}}} \tag{2}
\end{equation*}

where ${f_s}$ is the sampling rate of $X$. In this study we used 1-minute batches to compute the average cycle length. The batch length impacts the precision of the estimated ${l_x}$, where a smaller length yields precise estimations robust to fast changes in HR. However, this requires frequent executions of FFT thus increasing the overall runtime of the framework. On the other hand, there should be at least two complete cardiac cycles present in the batch to prevent detecting dominant frequencies of incomplete meta-subsequences.

**Fig. 2.**
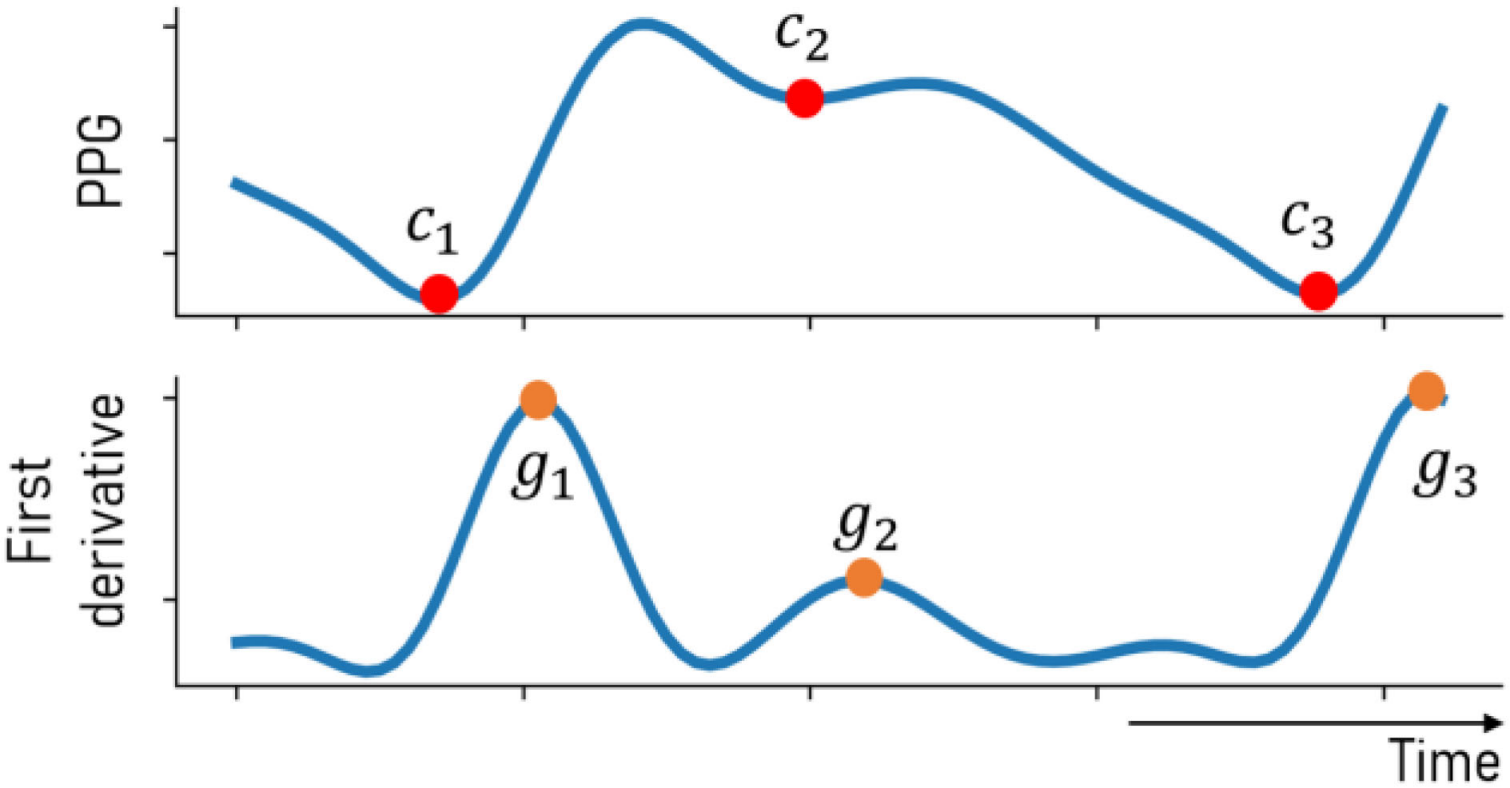
Candidate endpoints are identified in the raw PPG signal and are assigned a likelihood score based on the gradient of the following peak.

$P({{c_t}|{x_t}})$ is the likelihood that the sample, ${x_t}$, is a realistic, candidate endpoint for the type of waveform being analyzed based on understanding of the physiological processes that compose it. For our experiments we use PPG, where we understand that start and endpoints for all cardiac cycles can be characterized by the onset point that immediately precedes the systolic fiducial point. Therefore, the set of candidate endpoints, $C$, within a given batch $X$ includes all local minima and will be scored based on the steepness of the immediately following peak. (Discussion of cardiac behavior represented by PPG included in Supplementary I.A.) This is formulated accordingly:

\begin{equation*}
P\left({{c_t}|{x_t}} \right) = \ \frac{{{g_t} - \text{min}\left({X^{\prime}} \right)}}{{\max \left({X^{\prime}} \right) - \text{min}\left({X^{\prime}} \right)}} \tag{3}
\end{equation*}

where ${g_t}$ is the value of the max slope point represented in the first derivative as a peak point, as shown in Fig. 2. All values in the first derivative of PPG are scaled to where the maximum value is 1 and the minimum value is 0 where the greater the max slope of the onset following a candidate endpoint, the more likely that it is a true endpoint. Understanding of cardiac cycle endpoint features is the only prior domain-knowledge required for this framework, and this concept is generalizable to all types of physiological waveforms that measure blood flow.

$P({d({{x_t},{y_m}})})$ is the morphology-based likelihood – derived from DTW – that each sample ${x_t}$ in $X$ is a candidate endpoint based on the comparison of each sample in the input stream to all points in the template ${y_m}$ in $Y$, to compose a running DTW distance matrix, $D({X,Y})$. Background material on DTW included in Supplementary I.B. Since the Euclidean distance of each sample-to-sample comparison is augmented with the minimum distance most adjacent (or most recently computed) in the distance matrix, we can consider the distance function as causal, making the most recent comparison at any given $t$, $d({{x_t},{y_m}})$, to be the most representative metric for similarity between $X$ and $Y$ at that time. Furthermore, the smaller this distance value becomes, the more likely the region we are analyzing is a subsequence (or cardiac cycle). The likelihood score is computed as follows:

\begin{equation*}
P\left({d\left({{x_t},{y_m}} \right)} \right) = \ {e^{ - \gamma *\;d\left({{x_t},{y_m}} \right)}} \tag{4}
\end{equation*}

We leverage the exponential function as it is a monotonically increasing function that will penalize large $d({{x_t},{y_m}})$ distance values appropriately. We also use $\gamma $ as a scaling factor to normalize the distribution of likelihood scores to be easily comparable with $P({c_t}|{x_t})$, this scaling factor should be determined empirically while considering the scale of DTW distance values and the amount of influence which the resulting $P({d({{x_t},{y_m}})})$ should carry on the final prediction task.

**Fig. 3.**
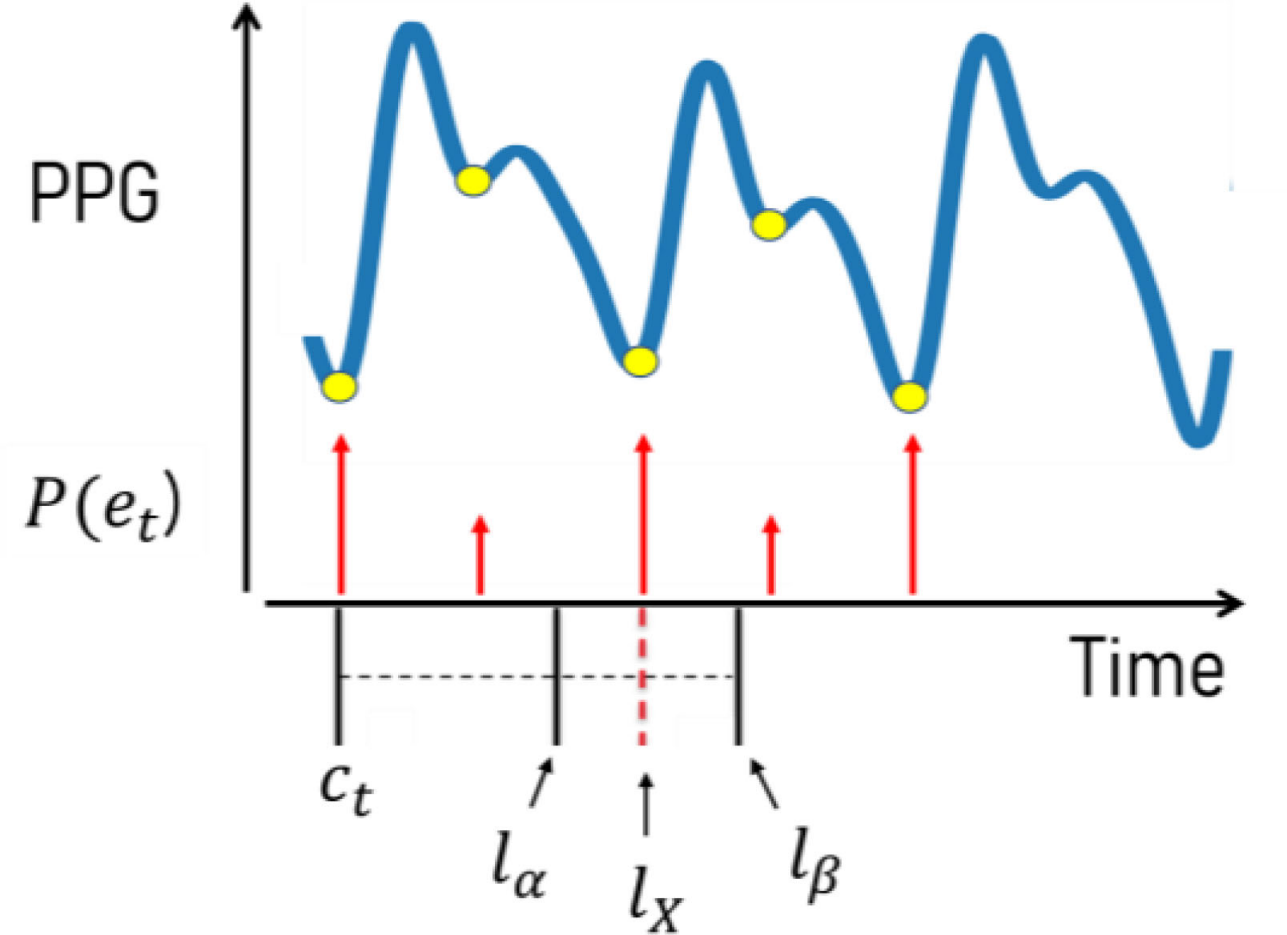
True cardiac cycle endpoints are identified as local maxima in $P({{e_t}})$ values within the search space constrained by an average cycle length, ${l_X}$, which is derived from local HR.

The search for true cardiac cycle endpoints occurs as DTW distances are computed. The process begins at the first potential ${c_t}$, where we expect a corresponding ${e_t}$ to exist around the next ${l_X}$ time steps of the stream with the greatest $P({{e_t}})$. By defining two generalizable parameters $\alpha $ and $\beta $, we can define the amount of tolerance surrounding ${l_X}$ that we will allow to detect an optimal ${e_t}$. Here, $\alpha *{l_X}$ will be the minimum distance in time steps after a potential ${c_t}$, therefore if another candidate endpoint occurs between the first candidate and ${l_\alpha } = \ {c_t} + \ \alpha *{l_X}$, then the segmentation process will reset and this new point will become the new potential cardiac cycle starting point. Then, $\beta *{l_X}$ will be the maximum distance after ${c_t}$ which a valid ${e_t}$ may exist, ${l_\beta } = \ {c_t} + \ \beta *{l_X}$, where we will accept the candidate endpoint with the greatest $P({{e_t}})$ between ${l_\alpha }$ and ${l_\beta }$ as the true ${e_t}$. If no candidate endpoint is detected in this region, then the algorithm will reset to the next candidate endpoint after ${l_\beta }$. Here, $\alpha $ and $\beta $ may be tuned to impact sensitivity of the framework but ideally the minimum distance should be large enough to avoid the possibility of false positives (such as detecting a dicrotic notch of the current cardiac cycle as ${e_t}$ in PPG), and the maximum distance should be greater than ${l_X}$ yet less than the potential location of non-endpoint fiducial points (such as the dicrotic notch in PPG) in the subsequent cardiac cycle. This approach may be visualized in Fig. 3.

### B. Automated Dynamic Template

We define the maximum number of templates allowed in the ensemble, $k$, and initialize it with a prime template, ${Y^*}$. Then, analysis of an input waveform batch begins with the prime template only. After feature extraction concludes for a region of the batch with a predefined length of $u$, if there are less than $k$ templates in the ensemble then a new template will be generated and added to it. This is accomplished by generating a consensus sequence based on the cardiac cycles detected in this region - using DTW barycenter averaging [23] – and its fiducial points will be annotated by the prime template. Further explanation of the composition of the consensus sequence is provided in Supplementary I.C. While multiple templates exist in the ensemble, each will conduct analysis over the region independently and the average warping path distance, $| {{w_{X,Y}}} |$, between each detected cardiac cycle and each template will be tracked. When all templates have completed their analysis, the template which yielded the lowest average $| {{w_{X,Y}}} |$ will be predicted as the optimal template, ${Y_{opt}}$, which has achieved the most accurate feature extraction for the set of detected cardiac cycles. Last, for the case that there are $k$ templates in the ensemble and ${Y^*} \ne {Y_{opt}}$, then an update will be triggered and the least frequently used template will be discarded from the ensemble. Otherwise, if ${Y^*} = {Y_{opt}}$, then an update will not be triggered. This template update protocol is possible due to the robustness of our cardiac cycle detection phase to at the least be capable of identifying the true start and endpoints despite that the fiducial point mapping could potentially be low-quality, where an additional pass over the most recent segment may be conducted with the newly generated template to achieve fiducial point identification with greater precision at the small cost of the additional time required to re-analyze the input batch.

## III. Results

**Fig. 4.**
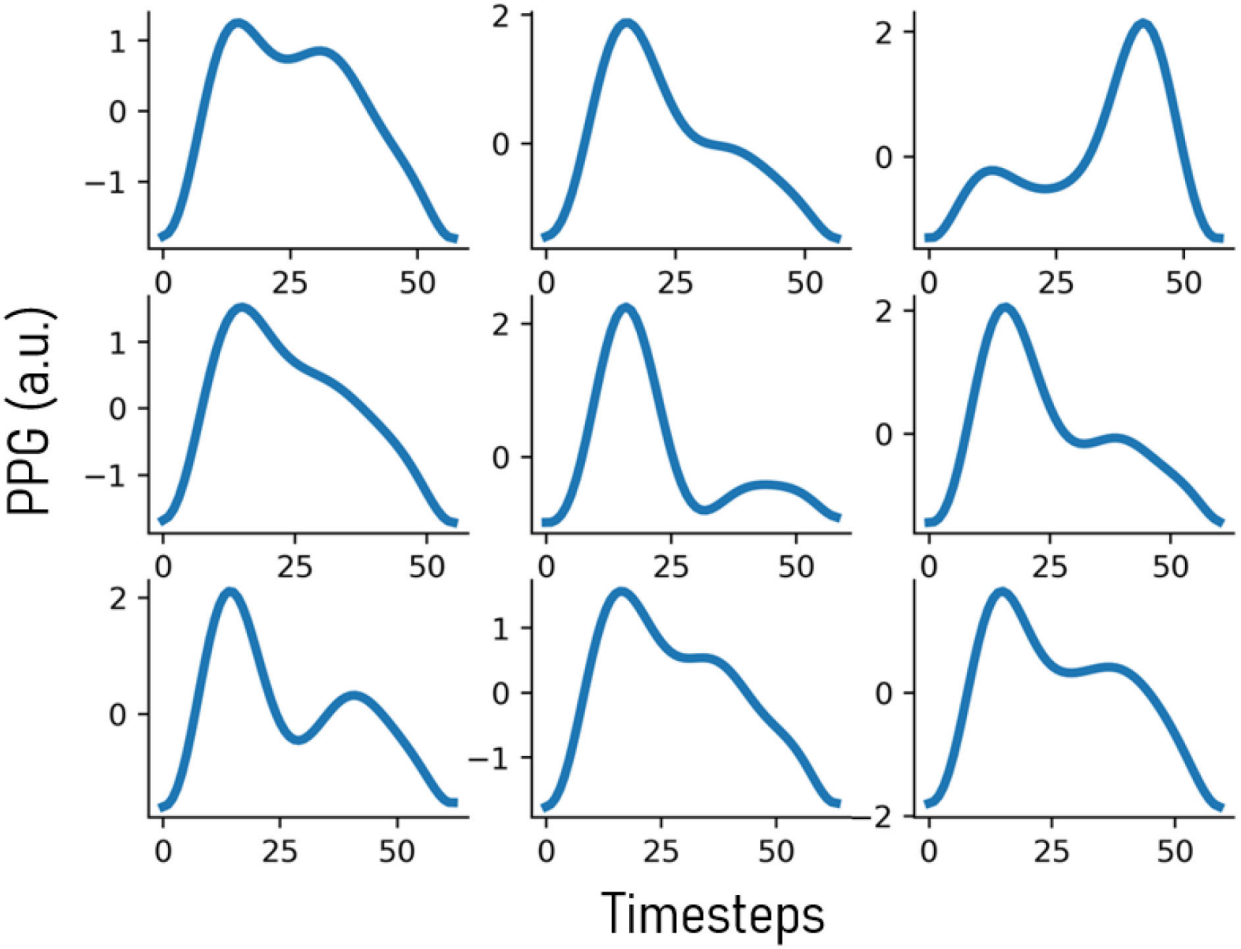
Non-exhaustive set of PPG cardiac cycle morphology variations that appear in the benchmark dataset.

We validate the effectiveness of Boosted-SpringDTW (single and dynamic template) to achieve comprehensive feature extraction from PPG waveforms that are impacted by respiration- and subject-induced morphological variations in the IEEE TBME Respiratory Rate Benchmark Data Set [24]. Particularly, in the previous work which introduced this dataset explains how PPG is impacted by respiration-induced variation with respect to amplitude (reflected pulse strength), intensity (changes in perfusion baseline), and frequency (effective HR) both within and across subject data – all of which require frequent adapting of algorithm thresholds to properly identify all waveform fiducial points. Furthermore, each subject's PPG data possesses a distinct waveform morphology that may also evolve over time. Such variations are best distinguished by the relationship amongst each fiducial point within a cardiac cycle. In Fig. 4 we visualize some prominent PPG morphological variations present in the IEEE TBME Respiratory Rate Benchmark Data Set. The scope of this study includes the proposed framework's ability to analyze variation in valid cardiac cycles’ waveform morphologies; therefore, we exclude instances severely corrupted by artifact noise such as those caused by motion. The dataset includes 42 participants – 29 children and 14 adults. Signals are sampled at 300 Hz, and the PPG signals were preprocessed with a 4^th^ order Butterworth Bandpass Filter with cutoff frequencies of [0.5, 5]. We empirically determined Boosted-SpringDTW parameters $\alpha $, $\beta $, and $\gamma $ to be set to 0.7, 1.3, and 5000. We compare performance to two baselines – the original SpringDTW algorithm [25] and adaptive thresholding. SpringDTW detects subsequence endpoints by tracking minimum $d({{x_t},{y_m}})$ distances over a stream. Adaptive thresholding leverages amplitude and distance-based heuristics to detect peak, slope, and valley features with PPG and its derivative signals. [26], [27]. We then evaluate IBI estimation with each of the identified fiducial points. To maintain a consistent problem setting in this study, we analyze only solutions that operate in the time-domain and that do not require training data to tune model parameters.

### A. Fiducial Point Identification

**Fig. 5.**
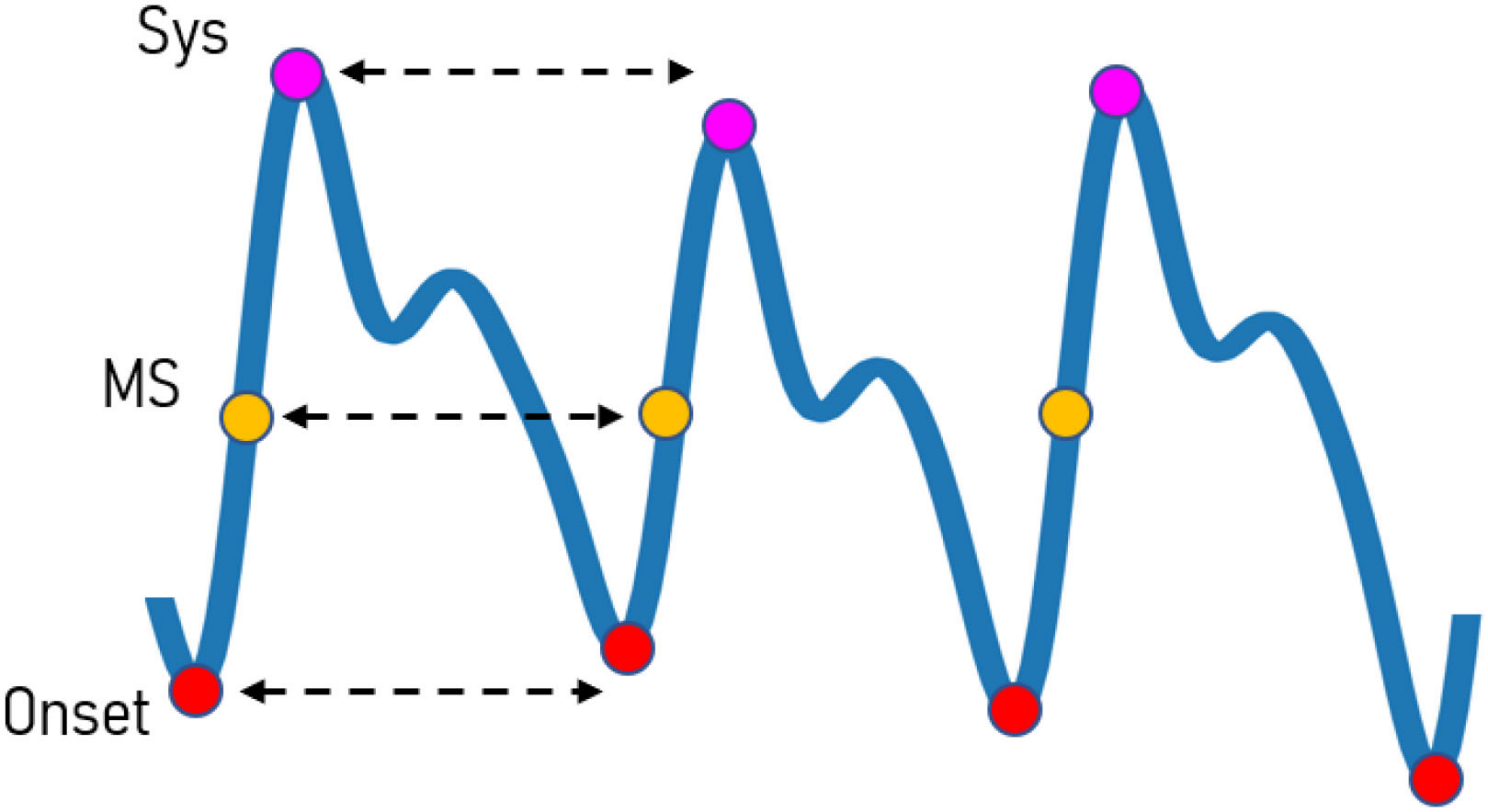
Fiducial points to be identified and used for IBI estimation. The onset points represent the start and end for each cardiac cycle, the max slope point gives insight into the rate of change in blood pressure as the heart contracts, and the systolic peak is directly correlated to the maximum pressure of the blood flow through this region of the body after it is pumped from the heart. Each of these fiducial points are commonly used in the estimation of several physiological parameters, including IBI, HR, HRV, BP, RR, and others.

Fig. 5 shows the fiducial points identified for this experiment that represent key events in the cardiac cycle [4]. We formulate both a classification and a regression task where each sample in a PPG stream is labelled as either a systolic peak (SYS), max slope point (MS), cardiac cycle endpoint (EP), or a non-fiducial point (NF); and precision is based on the reported timestamp. All fiducial points were manually labeled with the assistance of a python-based graphical user interface [28], yielding 27850 SYS points, 27843 MS points, 27926 EP points, 5964423 NF points. Since there exists a significant class imbalance between the number of NF points versus the number of SYS, MS, and EP points, we include evaluation metrics that balance positive predictions versus the types of negative predictions made by each model to reflect when a method is overpredicting for the majority class (NF points) [29]. The evaluation metrics include precision, recall, F1-score, and root mean squared error (RMSE) which are computed as follows

\begin{align*}
Precision =& \frac{{TP}}{{TP + FP}} \tag{5}\\
Recall =& \frac{{TP}}{{TP + FN}} \tag{6}\\
F1 =& 2 \cdot \frac{{Precision \cdot Recall}}{{Precision + Recall}} \tag{7}\\
RMSE =& \sqrt {\frac{1}{F}\mathop \sum \limits_{i = 1}^F {{\left({\widehat {{T_i}} - {T_i}} \right)}^2}} \tag{8}
\end{align*}

where TP is a correctly labelled fiducial point, TN is a correctly labelled non-fiducial point, FP is an incorrectly labelled non-fiducial point, FN is an incorrectly labelled fiducial point, $F$ is the number of fiducial point observations, ${T_i}$ refers to the ground truth timestamp of a fiducial point, and ${\hat{T}_i}$ refers to the predicted timestamp of a fiducial point. These metrics were computed for each class of fiducial points independently. When a given method is overpredicting for the NF class, the recall score will be low while a high recall score reflects the method's ability to distinguish between classes with high precision. RMSE only compares time distances for positive predictions. Therefore, the number of samples may vary for each algorithm. Table I shows the scores for fiducial point identification performance for each algorithm included in the study where Boosted-SpringDTW-ST refers to using a single template and Boosted-SpringDTW-DT refers to using the dynamic template.

**TABLE I:** Fiducial Point Identification Performance

	Sys				MS				EP				
	Prec	Rec	F1-Score	RMSE (ms)	Prec	Rec	F1-Score	RMSE (ms)	Prec	Rec	F1-Score	RMSE (ms)
SpringDTW	0.939	0.433	0.592	25.1	0.998	0.460	0.630	24.2	0.0	0.0	0.0	186.1
Adaptive Thresholding	0.996	0.789	0.880	42.6	0.994	0.790	0.881	44.3	0.688	0.995	0.813	166.9
Boosted-SpringDTW-ST	0.981	0.967	0.974	57.2	0.963	0.949	0.956	55.0	0.986	0.986	0.986	34.5
Boosted-SpringDTW-DT	0.995	0.980	0.987	34.9	0.979	0.965	0.972	36.2	0.986	0.986	0.986	34.5

### B. Physiological Parameter Estimation

We evaluate the estimation of a well-studied physiological parameter from the detected fiducial points, IBI, which is highly regarded for health monitoring [30]. IBI is defined as the time difference between consecutive heart beats and is computed as $IB{I_t} = Sy{s_t} - Sy{s_{t - 1}} = M{S_t} - M{S_{t - 1}} = {t_e} - {t_s}$. Therefore, since IBI is the beat-by-beat reflection of HR (where HR is typically measured as an average of consecutive cardiac cycles in a time window), analyzing IBI estimation quality grants a fine-tuned insight into how HR impacts key fiducial point identification. Ground truth IBI was extracted from the dataset's ECG waveform R peaks – also manually annotated. We evaluate the estimation performance using mean absolute error (MAE) in milliseconds (ms) and also using Pearson's correlation between the closest predicted IBI value in time and the ground truth measurements with a maximum difference in reported time of 1 second. We also included a plausibility filter for the estimated IBI values where estimates that implied HRs below 18 beats per minute (IBI of 300 ms) or above 90 beats per minute (IBI of 1500 ms) were discarded. This yields 25,874, 65,629, 79,451, and 79,464 valid IBI estimates for SpringDTW, adaptive thresholding, and the two proposed Boosted-SpringDTW frameworks. Results shown in Table II indicate that fiducial point identification performance is directly linked to the quality of the resulting estimates for physiological parameters. In addition, in Supplementary Tables I.E-I and I.E-II we analyze the IBI estimation performance by Boosted-SpringDTW-DT per subject to grant insight into the notion of inter- and intra-subject variability.

**TABLE II:** IBI Estimation Performance

	Sys		MS		EP		Valid
	MAE, ms (%)	Corr	MAE, ms (%)	Corr	MAE, ms (%)	Corr	Predictions, # (%)
SpringDTW	29.14 ± 1.93 (5.08)	0.823	31.84 ± 2.02 (5.53)	0.81	219.28 ± 9.93 (31.69)	-0.377	25,874 (66.8)
Adaptive Thresholding	131.61 ± 3.24 (23.91)	0.356	142.18 ± 3.27 (25.49)	0.335	110.64 ± 2.25 (13.44)	0.443	65,629 (87.1)
Boosted-SpringDTW-ST	10.81 ± 0.44 (1.44)	0.976	11.71 ± 0.41 (1.56)	0.979	11.41 ± 0.27 (1.59)	0.989	79,451 (99.9)
Boosted-SpringDTW-DT	7.59 ± 0.14 (1.09)	0.997	8.54 ± 0.15 (1.24)	0.996	11.41 ± 0.27 (1.59)	0.989	79,464 (99.9)

## IV. Discussion

Both versions of the proposed Boosted-SpringDTW framework show strong performance for identifying each class of fiducial points achieving scores over 0.949 for precision, recall, and F1. The improved F1 compared to the two baseline algorithms are the best reflection of overall performance since they represent the balance in TP, FP, and FN predictions. The dynamic template also yields an additional 1-2% improvement labelling SYS and MS points. Although the lowest RMSE values were by SpringDTW, it should be noted that RMSE reflects an average error of all predictions where this approach yielded far fewer positive predictions compared to each evaluated method for each fiducial point thus it was less susceptible to inherently noisy predictions.

**Fig. 6.**
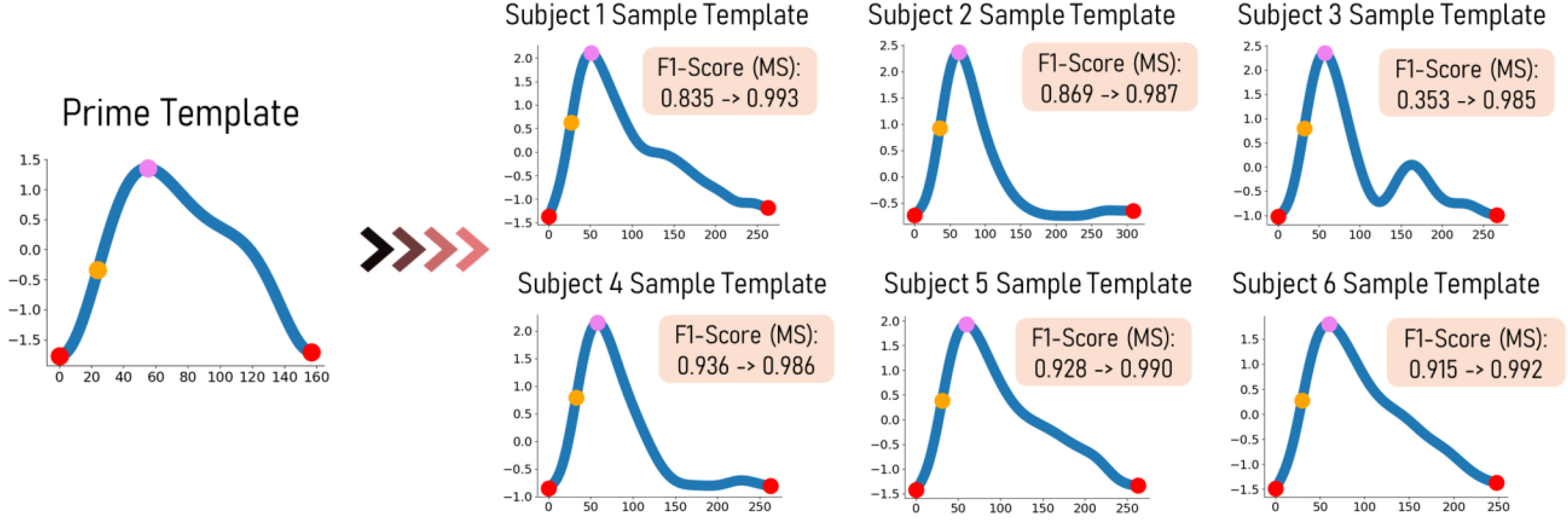
Generated dynamic templates for six subjects where the single template case achieved F1-scores below 0.95 for identifying MS yet the dynamic template is able to achieve scores greater than 0.98. The dynamic template update protocol precisely captures intricacies of evolving waveform morphology.

In Fig. 6 we show dynamic templates generated for six subjects where the single template framework previously achieved F1-scores less than 0.95 for identifying MS points yet we observe scores over 0.98 with the dynamic template. This is due to the dynamic template update protocol that adapts to new variations in waveform morphology.

**Fig. 7.**
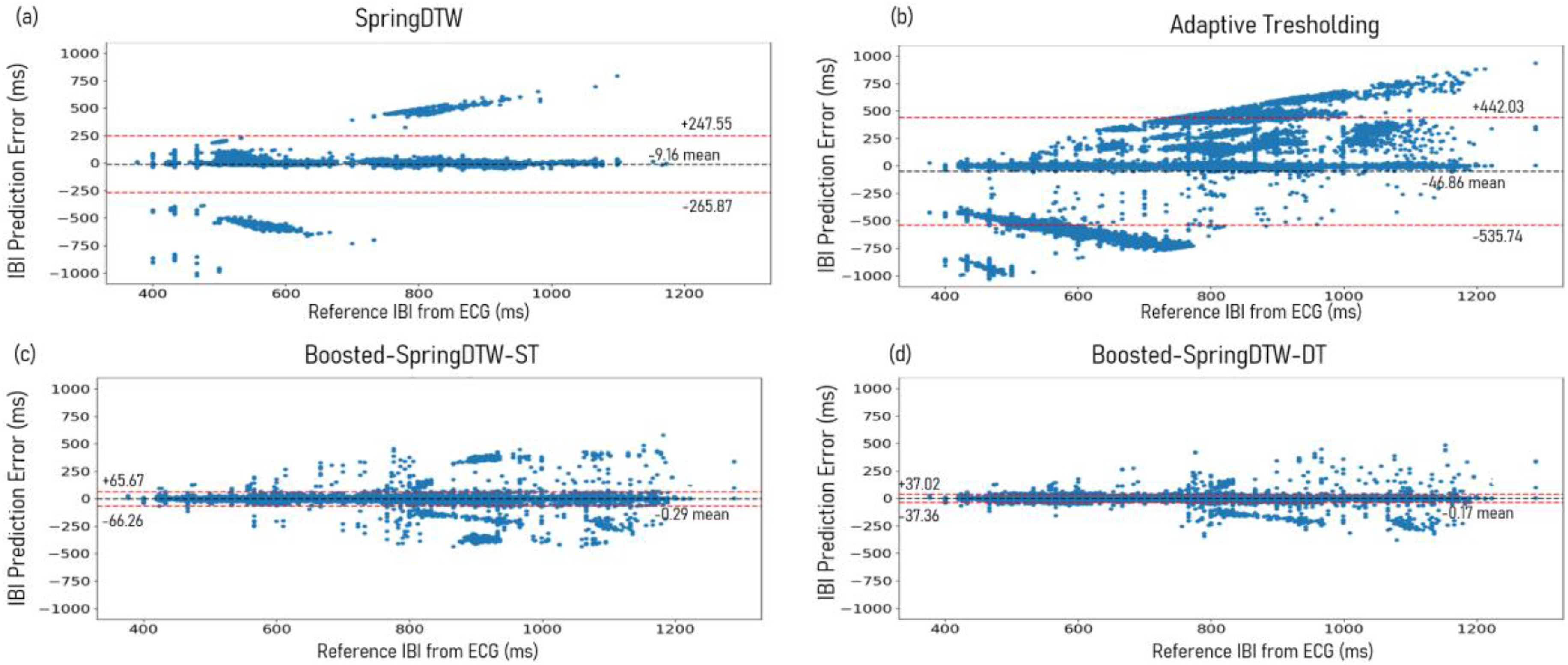
Difference plots evaluating IBI predictions computing using all fiducial points detected by (a) SpringDTW, (b) adaptive thresholding, (c) Boosted-SpringDTW-ST, and (d) Boosted-SpringDTW-DT.

The top performers for IBI estimation with each fiducial point are the two versions of Boosted-SpringDTW yielding MAE scores less than 12 ms and correlation coefficients greater than 0.98. We observe improved IBI estimation with SYS and MS points when Boosted-SpringDTW leverages the dynamic template, yielding a 28.5% improvement in MAE and a 2.0% improvement in the correlation coefficient values. In the difference plots shown in Fig. 7c and Fig. 7d, for all approaches we observe that error increases as IBI values become larger (indicating a decrease in HR), thus reflecting that as the HR drops it becomes more difficult to distinguish cardiac cycles. However, we observe a smaller margin of error for Boosted-SpringDTW compared to the two baseline approaches – observed in Fig. 7a and 7b – and noticeably less outlier predictions when using the dynamic template. Discussion on runtime analysis is in Supplementary I.D.

**Fig. 8.**
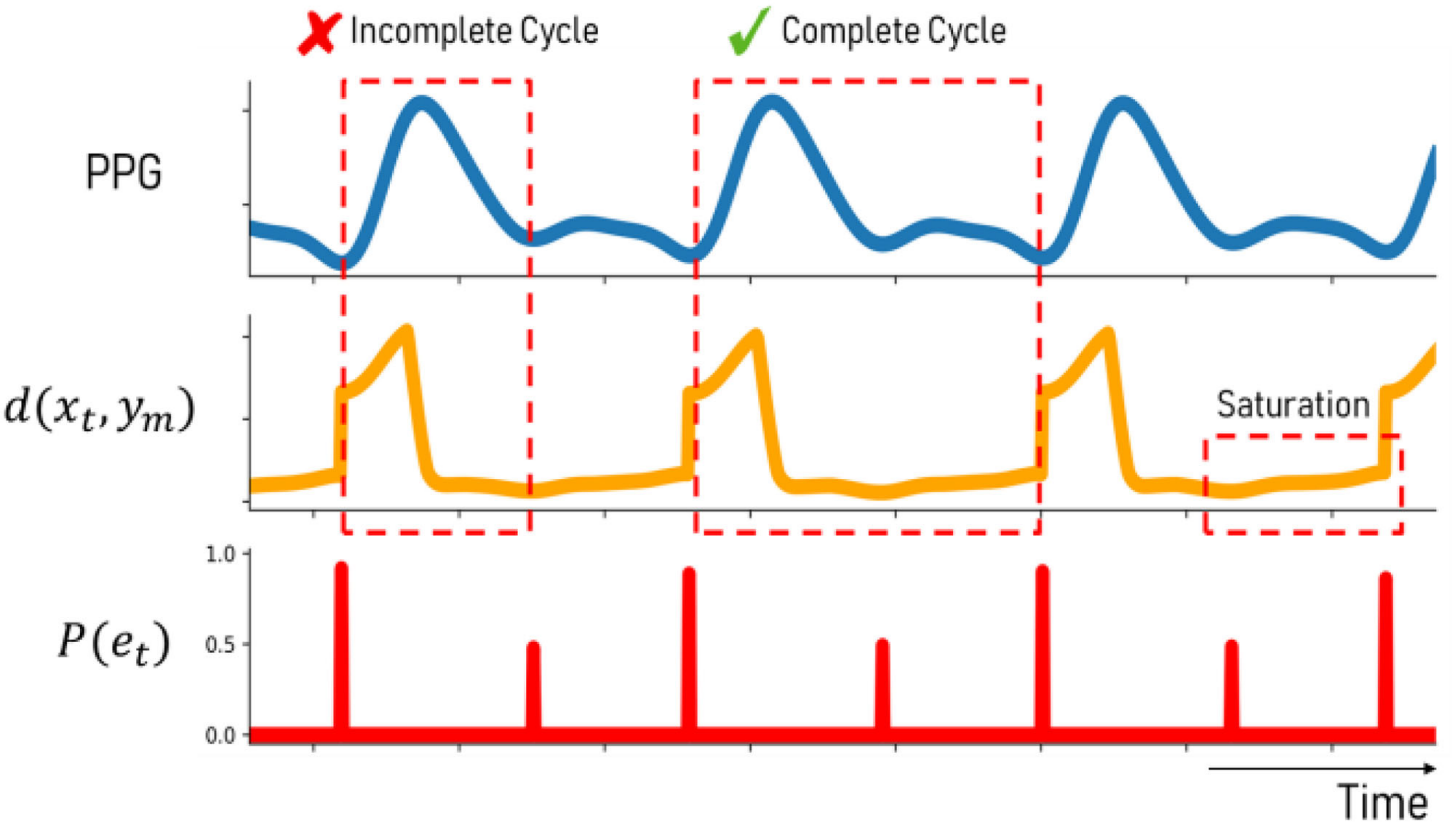
Challenges associated with $d({{x_t},{y_m}})$ trend saturation are overcome with the $P({{e_t}})$ endpoint likelihood score based on boosting.

Fig. 8 shows the trend of $d({{x_t},{y_m}})$ as it is computed over a PPG stream for each time step and the associated endpoint likelihood scores. The $d({{x_t},{y_m}})$ distances are greatest at the very beginning of a waveform but will reach the local minima when the first possible subsequence is encountered. However, despite the existence of more optimal subsequences, the $d({{x_t},{y_m}})$ values will saturate and gradually continue to increase since the distance at each subsequent step will accumulate over time. Since SpringDTW targets sequence matching by detecting local minima for $d({{x_t},{y_m}})\ $it may be prematurely detect incomplete cardiac cycles. Therefore, we show that Boosted-SpringDTW's likelihood scores better distinguish between a sub-optimal subsequence and an optimal cardiac cycle.

An immediate follow up work for this study would be to conduct rigorous stress testing to definitively evaluate the maximum levels of noise (such as motion noise) which Boosted-SpringDTW is able to handle. Also, extending the analysis to different modalities that may present new challenges would lead to further development of Boosted-SpringDTW capabilities. Furthermore, exploration into the value of this work to serve as a preprocessing tool contributing to end-to-end prediction frameworks. Particularly, Boosted-SpringDTW has the ability to segment and annotate physiological waveform segments that represent cardiac cycles which have been fed as input to prediction models pursuing tasks such as physiological parameter estimation [32]. Last, our framework is capable of providing a notion of waveform quality for each segmented cardiac cycle through its comparison to a template waveform – typically leveraged to measure prediction confidence or uncertainty and to be explored in future work.

## V. Conclusion

In this work, we proposed Boosted-SpringDTW to perform comprehensive feature extraction for remote health monitoring. We enable the use of DTW for segmentation of quasi-periodic signals and without the need for pre-defined thresholds by combining the strengths of simple, minimal domain-specific heuristics and the generalizable DTW signal analysis method. We overcame the challenges associated with morphological variations in PPG with the notion of a dynamic template. We validated performance by evaluating precision, recall, F1-score, and RMSE performance when attempting to identify SYS, MS, and EP points of a collected PPG signal induced with variation in signal morphology due to inter-subject variability and respiratory behaviors. The proposed framework achieved superior performance for the fiducial point identification task compared to the original SpringDTW implementation and to the standard adaptive thresholding approach. This led to superior IBI estimation by Boosted-SpringDTW frameworks.
